# A COF-based nanoplatform for highly efficient cancer diagnosis, photodynamic therapy and prognosis†
†Electronic supplementary information (ESI) available: Experimental details and supplementary figures. See DOI: 10.1039/d0sc00847h


**DOI:** 10.1039/d0sc00847h

**Published:** 2020-06-16

**Authors:** Peng Gao, Mengzhen Wang, Yuanyuan Chen, Wei Pan, Ping Zhou, Xiuyan Wan, Na Li, Bo Tang

**Affiliations:** a College of Chemistry , Chemical Engineering and Materials Science , Key Laboratory of Molecular and Nano Probes , Ministry of Education , Collaborative Innovation Center of Functionalized Probes for Chemical Imaging in Universities of Shandong , Institute of Molecular and Nano Science , Shandong Normal University , Jinan 250014 , P. R. China . Email: panwei@sdnu.edu.cn ; Email: lina@sdnu.edu.cn ; Email: tangb@sdnu.edu.cn

## Abstract

Covalent organic frameworks (COFs) have emerged as a kind of promising material for analytical and biomedical purposes. However, simultaneous cancer diagnosis and therapy with COFs remain a challenge. We report here a COF-based theranostic nanoplatform by integrating a dye-labeled oligonucleotide onto porphyrin-based COF nanoparticles for highly efficient cancer diagnosis and therapy. The fluorescence of the dye was effectively quenched by the COF through fluorescence resonance energy transfer (FRET). In the presence of biomarker survivin mRNA, more stable duplexes were formed and separated from the COF NPs, enabling the recovery of the fluorescence signal and selective cancer imaging. Under NIR laser irradiation, COF NPs generated abundant reactive oxygen species (ROS) to induce cancer cell apoptosis owing to their crystalline reticular structure. *In vitro* and *in vivo* experiments revealed that the nanoplatform has a high specificity and inhibition effect toward cancer cells and solid tumors. Interestingly, prognostic evaluation was also realized with COF-survivin. This work will offer new insights into COF-based probes and inspire the development of more versatile tools for biomedical applications.

## Abstract 2

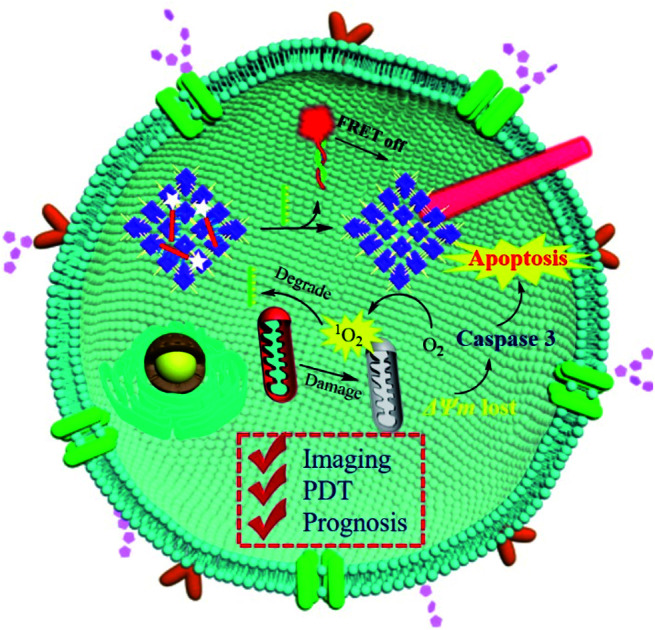
A covalent organic framework-based nanoplatform has been developed for cancer imaging, photodynamic therapy, and prognosis.

## Introduction

Covalent organic frameworks (COFs), as a class of emerging crystalline porous polymeric materials, possess tremendous potential for application in a variety of fields owing to their facile design and well-defined structure.^1^–^4^ Over the past dozen years, COFs with different chemical constituents and functionalities have been enthusiastically designed and employed for gas adsorption/separation, catalysis, optoelectronics, energy storage, environmental purposes and so on.^5^–^8^ COFs are also promising candidates for biomedical applications owing to their unique stability, biocompatibility and functional diversity.^9^–^12^ But the poor dispersibility and unsatisfactory bioavailability caused by their large size are still the major bottlenecks for COFs to be used for biomedical applications.^13^–^17^ Nanoscale COFs possessing better dispersibility and higher bioavailability are more suitable for biomedical purposes.

Photodynamic therapy (PDT), employing a specific laser to excite a photosensitizer (PS) to generate reactive oxygen species (ROS) and kill malignant cells, is highly spatiotemporally controlled and has been approved for clinical cancer treatment.^18^–^21^ Novel PSs are the bases for efficient cancer PDT. However, traditional PSs with large aromatic structures often suffer from poor solubility and tend to aggregate in physiological environments, which can significantly compromise their therapeutic effects.^22^,^23^ Nanoscale PSs with a regular porous structure could ensure high PS density and prevent unwanted aggregation, and they are ideal tools for highly efficient ROS generation.^24^–^26^ Thus, nanoscale COF-based PSs should be promising candidates for application in PDT.

Traditionally, cancer diagnosis and treatment are individual and separate processes.^27^–^32^ Because the metabolism pathways and biodistribution of diagnostic and therapeutic agents are different, it is difficult to timely and effectively guide the treatment plan based on the diagnosis result.^33^ Recently, theranostic probes that simultaneously integrate diagnostic and therapeutic functions have attracted considerable attention, owing to their potential in correctly adjusting the way of treatment and potentiating disease prognosis.^34^–^38^ Therefore, a COF-based theranostic nanoplatform will be highly desirable for cancer detection, treatment, and prognosis.

**Scheme 1:**
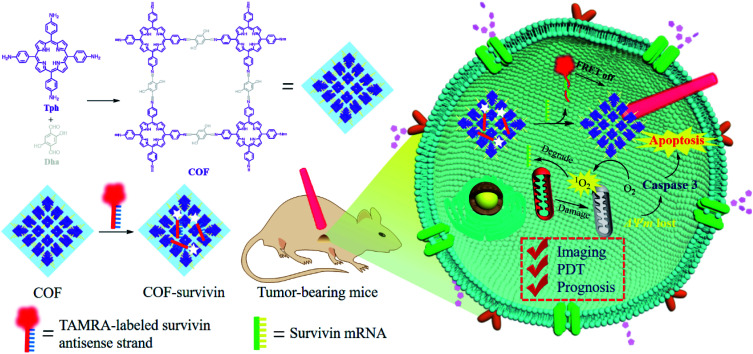
Schematic illustration of the preparation of theranostic nanoplatform COF-survivin for tumor imaging, PDT and prognostic evaluation applications.

Herein, we developed a nanoscale COF-based theranostic nanoplatform. A nanoscale porphyrin-based COF was prepared and a TAMRA-labeled survivin antisense strand (TSAS) was integrated onto the COF NPs to give rise to the nanoplatform (termed COF-survivin). The crystalline reticular structure endowed the COF with better stability and higher ROS generation ability in aqueous solution than those of the porphyrin monomer, while the large planar structure composed of the strong π-electron system makes it easy to absorb DNA single strands and quench the fluorophore to form a stable nanoplatform. The TSAS could readily form a duplex structure with survivin mRNA (a cancer biomarker, closely related to the genesis and development of several cancers),^39^,^40^ which significantly weakens the interaction between the TSAS and COF, resulting in the fluorophore being far away from the COF. Correspondingly, the fluorescence can be restored by the FRET prohibition, enabling specific cancer imaging. Further irradiation of COF-survivin with red light could generate abundant toxic ROS in cancer cells to induce oxidation stress, decrease the mitochondrial membrane potential (MMP), and trigger cell apoptosis. Based on this, COF-survivin was successfully employed for highly selective cancer cell/tissue imaging and efficient PDT. Interestingly, prognostic evaluation was also demonstrated (Scheme 1).

## Results and discussion

**Fig. 1:**
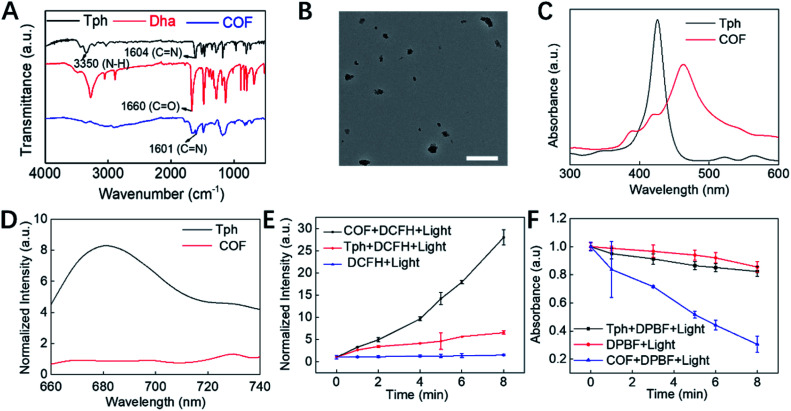
Structural and property characterization of the COF. (A) FT-IR spectra of Tph, Dha and the COF. (B) TEM image of the COF NPs. The scale bar is 500 nm. (C) UV-Vis adsorption and (D) the fluorescence spectra of Tph and COF NPs. Comparison of (E) the fluorescence enhancement of DCFH and (F) the absorption decay rate of DPBF caused by Tph and the COF.

COF NPs were prepared based on a solvothermal strategy.^16^ According to the FT-IR spectrum (Fig. 1A), the peak at 3350 nm refers to the N–H stretching band; the intensity of the C=O stretching band at 1660 nm is decreased, while a new peak at 1601 nm appeared corresponding to the C=N stretching band, demonstrating the successful condensation between amino and aldehyde groups. Moreover, multiple intense peaks were observed in the PXRD pattern, indicating the successful preparation of crystal COF structures (Fig. S1†). The TEM (Fig. 1B) and SEM (Fig. S2A†) results reveal that the obtained COF NPs have a uniform quasi spherical structure and good dispersibility. The UV-Vis spectrum (Fig. 1C) of COF NPs red shifted compared to that of the monomer, which should be ascribed to the formation of the strong π-electron system. The unique UV-Vis absorption effect makes COF NPs promising for constructing FRET-based probes. The fluorescence properties of COF NPs and the monomer were further investigated, and the reduced fluorescence (Fig. 1D) of COF NPs may enhance the ROS generation effect due to the restricted energy decay.^15^ Therefore, the ROS generation effect of COF NPs and the monomer was further evaluated. According to the results shown in Fig. 1E, the ROS generation by COF NPs was significantly higher than that by the porphyrin monomer in aqueous solution owing to the regular structure and reduced energy decay. Further investigation revealed that singlet oxygen (^1^O_2_), one of the most toxic ROS,^41^ was the main ROS generated by the COF NPs (Fig. 1F).

**Fig. 2:**
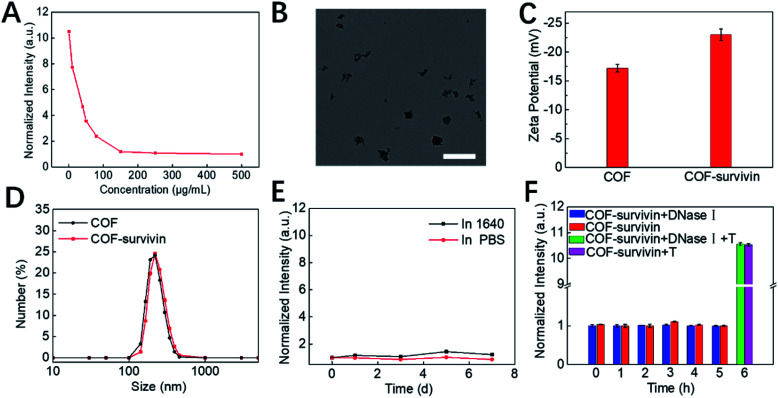
Preparation and characterization of COF-survivin. (A) Fluorescence quenching of the TSAS by different concentrations of COF NPs. (B) TEM image of COF-survivin. The scale bar is 500 nm. (C) Zeta potential and (D) DLS distribution of COF NPs and COF-survivin. (E) Relative fluorescence intensity changes of COF-survivin during incubation in PBS and DMEM for 0–7 d. (F) Relative fluorescence intensity changes of COF-survivin in the presence of DNase I and the survivin target (T).

Inspired by the unique structural characteristics of the obtained COF NPs, we assume that they could effectively adsorb DNA single strands and quench the fluorophore. By adding COF NPs into the TSAS solution, the fluorescence of TAMRA was quenched and reached a plateau as the concentration of COF NPs reached 150 μg mL^–1^ (Fig. 2A and S3†). Therefore, this ratio was selected to prepare COF-survivin for further investigations. The morphology of COF-survivin was similar to that of COF NPs (Fig. 2B and S2B†). The zeta potential of the COF NPs decreased from –17 to –22.5 due to the attachment of more negatively charged oligonucleotides (Fig. 2C). The DLS of COF NPs was slightly enhanced because of the successful DNA loading (Fig. 2D). Based on the fluorescence standard curve, the DNA loading amount on the COF was calculated to be 0.572 nmol mg^–1^ (Fig. S4†). Further investigation demonstrated that the obtained nanoplatform has a good storage stability (Fig. 2E) and anti-DNase I effect (Fig. 2F), mostly due to the formation of strong hydrogen bonds and π–π stacking interaction between the DNA chain and COF NPs, which prevented non-specific DNA release and reduced the interaction between DNase I and DNA molecules. Further addition of the survivin target into the COF-survivin solution could generate stable duplexes, which could reduce the interaction between the TSAS and COF NPs and restore the fluorescence by FRET prohibition (Fig. 2F and S5†). Therefore, COF-survivin is promising for simultaneous cancer imaging and PDT.

**Fig. 3:**
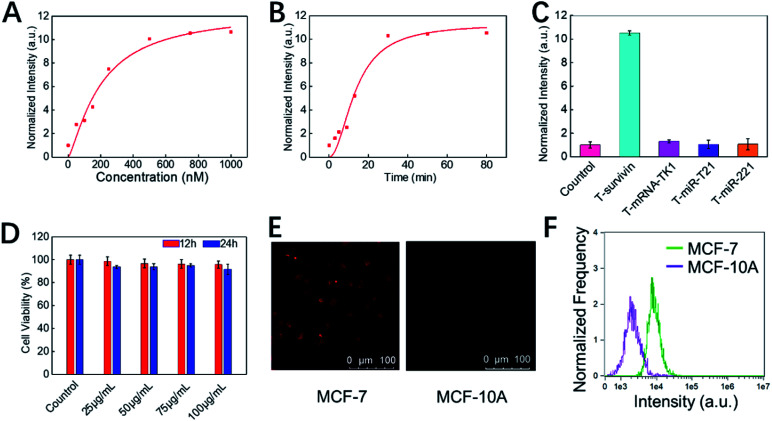
*In vitro* detection performance of COF-survivin. (A) Fluorescence intensities of COF-survivin in the presence of various concentrations of survivin targets (0–1000 nM). (B) Time-dependent fluorescence changes of COF-survivin in the presence of the survivin target (500 nM). (C) The responses of COF-survivin to different targets (500 nM). (D) Cell viability of MCF-7 cells after incubation with different concentrations of COF-survivin for 12 and 24 h. (E) Confocal images of MCF-7 and MCF-10A cells treated with COF-survivin. (F) Flow cytometry analysis of the cells treated with COF-survivin.

Next, the *in vitro* detection performance of COF-survivin was further investigated. According to the fluorescence spectra, the fluorescence of the TSAS could be completely restored by adding the survivin target into the probe solution, and the response time was less than 30 min (Fig. 3A, B, S6 and S7†). Furthermore, benefitting from the rule of complementary base pairing, COF-survivin has excellent target specificity; therefore its fluorescence signal can only be recovered in the presence of T-survivin (Fig. 3C). In order to be used for biomedical purposes, the biocompatibility of probes is highly important. We investigated the dark toxicity of COF-survivin with MTT assay (Fig. 3D). The cells maintained high viability even when incubated with COF-survivin at a concentration of 100 μg mL^–1^ for 24 h, implying that the developed theranostic nanoplatform is non-toxic to living cells under dark conditions. Therefore, we further evaluated the *in vitro* diagnostic effect of COF-survivin. Interestingly, by incubating COF-survivin with MCF-7 and MCF-10A cells, the cancer cell line was specifically lit up because survivin was overexpressed, much brighter than the normal cell line (Fig. 3E). The above phenomenon was also demonstrated by quantitative flow cytometry analysis (Fig. 3F). The reliability of the probe was further identified on A549/Beas-2b cell lines, which also demonstrated that COF-survivin could be used for survivin overexpressed cancer cell imaging. (Fig. S8†) Therefore, the COF-based nanoplatform could be employed for cancer cell diagnosis.

**Fig. 4:**
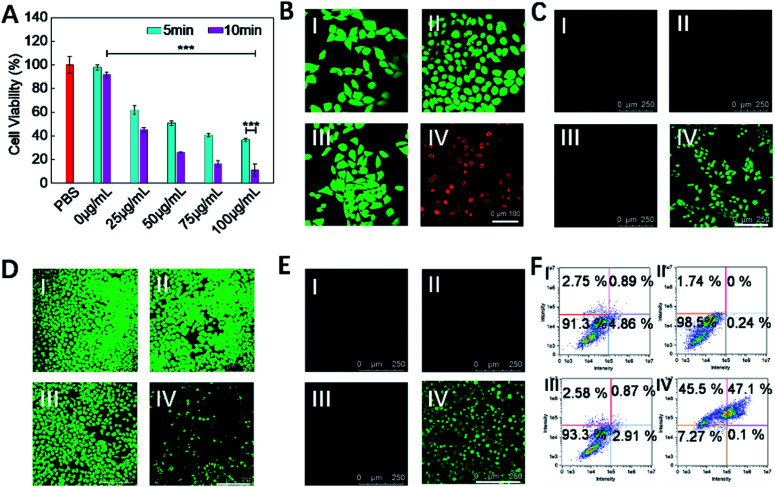
*In vitro* therapeutic performance of COF-survivin. (A) Cell viabilities of MCF-7 cells treated with different concentrations of COF-survivin followed by 5/10 min laser irradiation. (B) Live/dead cell staining assay, Scale bar = 100 nm. (C) Detection of intracellular ROS with DCFH-DA, scale bar = 250 nm. (D) Evaluation of Δ*Ψ*_m_ with Rhodamine 123, scale bar = 250 nm. (E) Immunofluorescence staining of intracellular caspase-3 expression with different treatments, scale bar = 250 nm. (F) Flow cytometry analysis of cell apoptosis following different treatments. The concentration of COF-survivin for cancer cell treatment was 100 μg mL^–1^, the laser power was 0.2 mW cm^–2^, and irradiation duration was 10 min. I: PBS only, II: PBS + laser, III: COF-survivin, IV: COF-survivin + laser (***p < 0.001).

Subsequently, the *in vitro* therapeutic effect of COF-survivin was studied. As shown in Fig. 4A, cells still maintained high viability after being irradiated with the laser alone, implying that the selected power and exposure time were non-injurious to living cells. However, obvious concentration- and time-dependent toxicity was observed on laser irradiated COF-survivin treated cells, implying the excellent PDT effect of the nanoplatform. Live/dead cell staining assay further proved the excellent cancer cell inhibition effect of COF-survivin (Fig. 4B). We further carried out a series of experiments to investigate the therapeutic mechanism of COF-survivin. As shown in Fig. 4C, obvious green fluorescence was observed in laser irradiated COF-survivin pretreated cells, revealing efficient ROS accumulation in this group; while no ROS accumulation was detected in the other groups, demonstrating that the nanoplatform possesses high biocompatibility and the laser parameters were feasible. This result was also proved by quantitative flow cytometry analysis. (Fig. S9†) Overexpression of ROS in living cells may cause oxidative stress and induce mitochondrial damage, which could further trigger cell apoptosis. Based on the Rhodamine 123 probe, the mitochondrial membrane potential (Δ*Ψ*_m_) of different groups was determined. Prominent Δ*Ψ*_m_ loss was detected in the therapy group as shown in Fig. 4D. The expression of apoptosis biomarker caspase-3 in cancer cells with different treatments was detected by immunofluorescence imaging assay. The results revealed obvious capase-3 activation in the cells in the therapy group (Fig. 4E). Flow cytometry analysis of cell apoptosis demonstrated that most cells survived in the control groups, while only 7.27% living cells were left in the therapy group (Fig. 4F). Taken together, it was practicable to employ COF-survivin for highly efficient cancer theranostics.

**Fig. 5:**
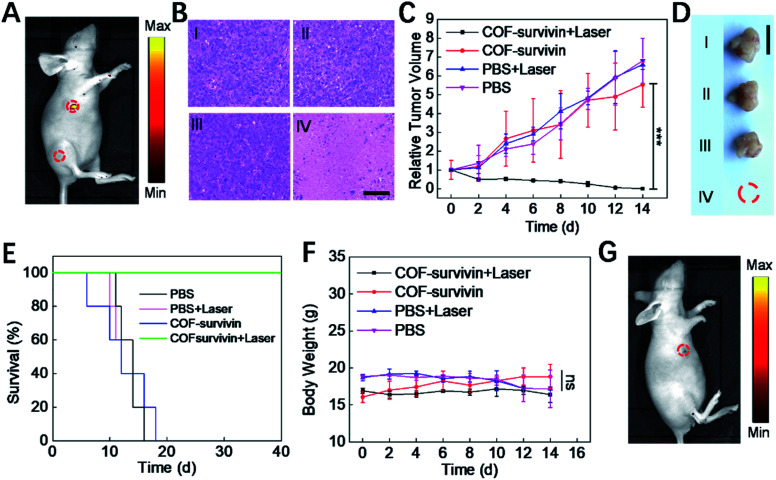
*In vivo* theranostic performance and prognostic evaluation of COF-survivin. (A) *In vivo* fluorescence imaging of tumor-bearing nude mice. 5 μL of COF-survivin (10 μg mL^–1^) was injected into the tumor tissue and leg muscle tissue as marked. (B) H&E staining of tumor tissue on the 3rd day post different treatment. Relative tumor volume (C) and representative tumor photograph on the 14th day (D) of different groups, and the scale bar = 1 cm. (E) The survival curves of tumor-bearing mice with different treatments. (F) The body weight change curve of mice receiving different treatments within 14 days. (G) *In vivo* fluorescence imaging of mice receiving COF-survivin + laser treatment on the 7th day, and 5 μL of COF-survivin (10 μg mL^–1^) was subcutaneously injected into the treatment area. I: PBS only, II: PBS + laser, III: COF-survivin, and IV: COF-survivin + laser. (***p < 0.001, ns, no significance).

To further evaluate the theranostic application potential of COF-survivin, a series of *in vivo* experiments were carried out. We first investigated whether the nanoplatform could differentiate tumor tissue from normal tissue. The result of *in vivo* fluorescence imaging (Fig. 5A) revealed that COF-survivin could effectively light up tumor tissue, while no fluorescence signal was observed from the normal tissue injected with the nanoplatform. Thus, COF-survivin could also efficiently discriminate the survivin expression levels in different tissues of living bodies. Subsequently, *in vivo* tumor PDT effect of the nanoplatform was further investigated. From the tumor H&E staining assay (Fig. 5B), obvious chromatin condensation could be observed in the tumor tissue injected with COF-survivin and treated with a 633 nm laser, while no pathological injury could be observed in the samples of the other three groups. An excellent tumor inhibition effect was realized and the tumor in the mice of the therapy group was completely eradicated (Fig. 5C and D). *In vivo* PDT with COF-survivin significantly prolonged the survival rate of tumor-bearing nude mice. All the mice survived over 40 days, while the mice in the control groups were all dead within 18 days (Fig. 5E). Further investigations revealed that the body weight of all the mice did not change obviously (Fig. 5F), and no damage could be observed in the main organs. (Fig. S10†) Therefore, COF-survivin was highly biocompatible and suitable for *in vivo* PDT. Survivin is an anti-apoptotic protein, and intracellular survivin levels are closely related to the genesis and development of tumors. Since PDT can significantly oxidize intracellular nucleic acids, further imaging of intratumoral survivin mRNA levels with the COF-based nanoplatform post treatment may help monitor the therapeutic effect to timely guide therapy, which is also known as the prognostic effect of theranostic probes. Therefore, on the 7th day post treatment, the mice of the therapy group were further treated with COF-survivin for *in vivo* fluorescence imaging. Interestingly, no fluorescence signal could be observed, indicating that *in vivo* PDT with the nanoplatform successfully eliminated the malignant tissue (Fig. 5G). All the *in vivo* experiments demonstrated that COF-survivin was a reliable theranostic nanoplatform for highly efficient *in vivo* tumor imaging, PDT and prognosis.

## Conclusions

In summary, we developed a COF-based theranostic nanoplatform (COF-survivin) by integrating a TAMRA-labeled survivin antisense oligonucleotide onto a nanoscale porphyrin-based COF. The crystalline structure characteristics of the COF endowed the nanoplatform with excellent stability and ROS generation capability. In the presence of cancer biomarker survivin mRNA, the readily formed duplex detached from the COF, which could restore the fluorescence by FRET prohibition and realize selective cancer imaging. Under NIR laser irradiation, the COF could generate abundant ^1^O_2_ species to induce cancer cell apoptosis, while no obvious dark toxicity was detected. The COF-based theranostic nanoplatform was successfully used for cancer diagnosis, therapy and prognostic evaluation. In view of the excellent designability and multifunctionality of the COF, many other targeting, diagnostic and therapeutic strategies could be rationally integrated to construct versatile COF-based theranostic probes. Compared with theranostic probes based on other nanomaterials such as metal–organic frameworks, this COF-based nanoplatform is heavy-metal free, relatively stable, biocompatible and highly integrated and performs well both *in vitro* and *in vivo*. This work opens up new avenues for COF-based probes and will inspire the use of more available tools for the biomedical field.

## Ethical statement

All the animal experiments were conducted and agreed with the Principles of Laboratory Animal Care (People's Republic of China) and the Guidelines of the Animal Investigation Committee, Biology Institute of Shandong Academy of Science, China.

## Conflicts of interest

The authors declare no competing financial interest.

## Supplementary Material

Supplementary information
