# Supplementary material for: A COF-based nanoplatform for highly efficient cancer diagnosis, photodynamic therapy and prognosis
Source: Chem Sci. 2020 Jun 16;11(26):6882–8. doi: 10.1039/d0sc00847h (PMC7500084; doi:10.1039/d0sc00847h)
Supplement: Supplementary file 1 [file SC-011-D0SC00847H-s001.pdf]

Electronic Supplementary Material (ESI) for Chemical Science.
This journal is © The Royal Society of Chemistry 2020

Supplementary information

A COF-based Nanoplatform for Highly Efficient Cancer Diagnosis, Photodynamic Therapy and Prognosis

Peng Gao,‡ Mengzhen Wang,‡ Yuanyuan Chen, Wei Pan, Ping Zhou, Xiuyan Wan, Na Li,* and Bo Tang**

College of Chemistry, Chemical Engineering and Materials Science, Key Laboratory of Molecular and Nano Probes, Ministry of Education, Collaborative Innovation Center of Functionalized Probes for Chemical Imaging in Universities of Shandong, Institute of Molecular and Nano Science, Shandong Normal University, Jinan 250014, P. R. China.
E-mail: panwei@sdnu.edu.cn; lina@sdnu.edu.cn; tangb@sdnu.edu.cn
‡. The authors contributed equally to this work.

Reagents and Materials.

5,10,15,20-tetrakis(4-aminophenyl)-21H,23H-porphine (Tph) and 2,5-dihydroxyterephthalaldehyde (Dha) were obtained from Changchun Third Party Pharmaceutical Technology Co. Ltd. DNA oligonucleotides were synthesized and purified by Sangon Biotechnology Co., Ltd (Shanghai, China) and the sequences of these oligonucleotides are shown in Table S1. Deoxyribonuclease I (DNase I) was purchased from Solarbio Science and Technology (Beijing, China). Rhodamine 123, 3-(4,5-dimethylthiazol-2-yl)-2,5- diphenyltetrazolium bromide (MTT) were purchased from Sigma Chemical Company. Calcein-AM/PI Double Stain Kit, Reactive Oxygen Species Assay Kit (DCFH-DA) and Annexin V-FITC Apoptosis Detection Kit were purchased from Beyotime (Nantong, China). Primary antibody for caspase-3 and FITC-labeled secondary antibody were purchased from Wuhan Boster Biological Technology Co., Ltd. (Wuhan, China). Confocal dish was purchased from Cellvis, Mountain View, CA. The human breast cancer cell line (MCF-7), normal immortalized human mammary epithelial cell line (MCF-10A) and HepG2 cell were purchased from Procell (Wuhan, China). All the other chemical reagents were of analytical grade and used without further purification.

Apparatus.

Fourier infrared spectrometer (Nicolet iS50 FT-IR) was used to characterize the infrared spectrum. Powder X-ray diffraction (XRD) pattern was obtained on a Rigaku SmartLab SE X-Ray Powder Diffractometer with Cu Kα line focused radiation ($\lambda = 1.5405$ Å). Transmission electron microscopy (TEM, HT7700, Japan) was carried out to characterize the morphology of the nanoparticles. Scanning electron microscopy (SEM) micrographs were recorded on a Hitachi SU8010 Scanning Electron Microscope. UV-vis spectroscopy was achieved with UV-1700 (Shimadzu, Japan). Fluorescence spectra were obtained using a FLS-980 Edinburgh Fluorescence Spectrometer with a Xenon lamp. The absorbance was measured in a microplate reader (Synergy 2, Biotek, USA) for the MTT assay. Confocal fluorescence imaging studies were performed using a TCS SP8 confocal laser scanning microscope (Leica, Germany). All pH measurements were performed with a digital pH-meter (pH-3e, LeiCi, China). Imaging flow cytometry was accomplished on Amnis ImageStream MarkII (Merck Millipore, Seattle, WA). *In vivo* fluorescence images were captured using live animal imaging system (IVIS Lumina III, US).

Preparation of COF NPs.

The porphyrin COF was prepared by mixing Dha (10 mg, 0.06 mmol) and Tph (20.3 mg, 0.03 mmol) in dichlorobenzene/butyl alcohol/6 M acetic acid (5/5/1, v/v/v, 1.7 mL). After sonication for 10 min, the mixture was degassed in a Pyrex tube (20 mL) through freeze-pump-thaw cycles for three times and then sealed off. The tube was heated 3 days at 120 °C. After that, the product was collected and washed with THF, acetone. The COF NPs was prepared by disperse COF into DMF followed by sonication for 30 min and further collected *via* differential centrifugation (4000 rmp to remove large COF and 10000 rmp to collect nanoscale COF NPs).

Fluorescence spectra of Tph and COF NPs.

Tph ethanol solution and COF NPs aqueous solution were prepared, the concentration of Tph were fixed to be 1 μg/mL. The fluorescence properties of Tph and COF NPs were further recorded with a fluorescence spectrophotometer.

ROS detection with DCFH.

To compare the ROS generation effects of Tph and the COF NPs (the concentration of Tph were fixed to be 1 μg/mL), DCFH was added to the solutions, respectively, and the solutions were

irradiated with 633 nm laser, the fluorescence properties of the solutions at different time points were recorded. Ex= 488 nm, Em=525 nm.

$^1O_2$ detection with ABMD.

To detect the $^1O_2$ generation effect of Tph and the COF NPs, ABMD was added into the solutions (the concentration of Tph were fixed to be 1 μg/mL), after irradiated with 633 nm laser with different times, the UV-Vis of the supernates were detected with UV/Vis spectrophotometer.

Preparation of COF-survivin.

COF-survivin was prepared by directly mixing TSAS solution with COF NPs. For optimizing the concentration ratio of TSAS and COF NPs, TSAS was mixed with different concentration of COF NPs (0, 10, 40, 50, 80, 150, 250 and 500 μg/mL). the obtained solutions were further stirred for 10 min and then the fluorescence intensities of the solutions were recorded with fluorescence spectrophotometer.

Storage stability.

To evaluate the storage stability of COF-survivin, COF-survivin was dissolved in PBS solution and 1640 solution, the fluorescence properties of the solutions were recorded for a week.

Kinetics Assay.

500 nM survivin target was added into the COF-survivin buffer solution (200 μg/mL), then the fluorescence signal of the solution at different timepoints were recorded with a fluorescence spectrophotometer.

mRNA-survivin Detection and Specificity Experiments.

To evaluate the detection and specificity of COF-survivin, different targets including T-survivin, T-mRNA TK1, T-miR21 and T-miR221 were added into the COF-survivin solution. After 30 min's incubation, the fluorescence properties of the solutions were recorded. with a fluorescence spectrophotometer.

Nuclease Assay.

To evaluate the nuclease stability of COF-survivin, DNase I was incubated with the probe directly. At different timepoints of 1, 2, 3, 4 and 5 h, the fluorescence property of the solution was detected and compared with COF-survivin solution. At 5.5 h, target survivin was further added into the two solutions and the fluorescence intensities were recorded after 30 min.

Cytotoxicity Assay.

To investigate the biocompatibility of the probe, MTT assay was carried out. MCF-7 cells were dispersed within 96-well microtiter plates at 37 °C for 24 h. After that, different concentrations of COF-survivin were incubated with the cells for 12 and 24 h, respectively. Then, the MTT solution (150 μL, 0.5 mg/mL in PBS) was further added to each well for another 4 h. After removing the remaining MTT medium, 150 μL of DMSO was added to each well. The absorbance was measured at 490 nm with microplate reader.

Confocal Fluorescence Imaging.

MCF-7, MCF-10A, A549, Beas-2b cells were cultured in confocal dishes. Then COF-survivin dissolved in culture medium was added into the dishes until the final concentration reached 100 μg/mL, the cells were further incubated for 4 h. Finally, the cells were washed with PBS and further imaged on a laser scanning confocal microscope.

Flow Cytometry.

MCF-7 and MCF-10A cells were cultured in cell dishes. Then, COF-survivin dissolved in DMEM was added into the dishes until the final concentration of COF-survivin reached 100 μg/mL. After

incubation for another 4 h, all the cells were digested and washed with PBS. Finally, the cells were analyzed on a flow cytometer.

In vitro PDT effect.

To evaluate the PDT effect of the probe, MTT assay was carried out. MCF-7 cells were dispersed within 96-well microtiter plates at 37 °C for 24 h. After that, different concentrations of COF-survivin were incubated with the cells for 4 h. Then, 633 nm (0.2 W/cm^2) laser was employed to irradiate the cells for 5 or 10 min. After added fresh DMEM solution, the cells were incubated for another 12 h. Then the MTT solution (150 μL, 0.5 mg/mL in PBS) was further added to each well for another 4 h. After removing the remaining MTT medium, 150 μL of DMSO was added to each well. The absorbance was measured at 490 nm with microplate reader.

Live/dead cell staining assay.

MCF-7 cells were cultured in confocal dishes for 24 h. The cells were divided into 4 groups: I: PBS only, II: PBS+Laser, III: COF-survivin, IV: COF-survivin+Laser. For COF-survivin containing groups, COF-survivin dissolved in DMEM was added into the dishes until the final concentration of COF-survivin reached 100 μg/mL, the cells were further incubated for 4 h. For laser irradiation groups, 655 nm laser (0.2 W/cm^2) was employed to irradiate the cells for 10 min. All the cells were further cultured for another 6 h. Finally, all the cells were stained with live/dead cell staining assay kit and further imaged on a laser scanning confocal microscope.

ROS imaging.

MCF-7 cells were cultured in confocal dishes for 24 h. The cells were divided into 4 groups: I: PBS only, II: PBS+Laser, III: COF-survivin, IV: COF-survivin+Laser. For COF-survivin containing groups, COF-survivin dissolved in DMEM was added into the dishes until the final concentration of COF-survivin reached 100 μg/mL, the cells were further incubated for 4 h. For laser irradiation groups, 633 nm laser (0.2 W/cm^2) was employed to irradiate the cells for 10 min. All the cells were preincubated with 1 mL DMEM containing 1 μL of DCFH-DA for 30 min before laser irradiation, and the cells were further cultured for another 6 h after different treatments. Finally, the cells were washed with PBS solution and further imaged on a laser scanning confocal microscope.

MMP detection.

MCF-7 cells were cultured in confocal dishes for 24 h. The cells were divided into 4 groups: I: PBS only, II: PBS+Laser, III: COF-survivin, IV: COF-survivin+Laser. For COF-survivin containing groups, COF-survivin dissolved in DMEM was added into the dishes until the final concentration of COF-survivin reached 100 μg/mL, the cells were further incubated for 4 h. For laser irradiation groups, 633 nm laser (0.2 W/cm^2) was employed to irradiate the cells for 10 min. All the cells were further cultured for another 6 h. Finally, all the cells were stained with Rhodamine 123 for 10 min and further washed with PBS. Finally, the cells were imaged on a laser scanning confocal microscope.

Immunofluorescence staining of caspase-3.

HepG2 cells were cultured in confocal dishes for 24 h. The cells were divided into 4 groups: I: PBS only, II: PBS+Laser, III: COF-survivin, IV: COF-survivin+Laser. For COF-survivin containing groups, COF-survivin dissolved in DMEM was added into the dishes until the final concentration of COF-survivin reached 100 μg/mL, the cells were further incubated for 4 h. For laser irradiation groups, 655 nm laser (0.2 W/cm^2) was employed to irradiate the cells for 10 min. All the cells were further cultured for another 6 h. Then, the cells were fixed with 4% paraformaldehyde for 20 min,

and stained with antibodies against caspase-3. Finally, the cells were washed with PBS and imaged on a laser scanning confocal microscope.

Flow cytometry analysis of apoptosis.

MCF-7 cells were cultured in cell dishes for 24 h. The cells were divided into 4 groups: I: PBS only, II: PBS+Laser, III: COF-survivin, IV: COF-survivin+Laser. For COF-survivin containing groups, COF-survivin dissolved in DMEM was added into the dishes until the final concentration of COF-survivin reached 100 μg/mL, the cells were further incubated for 4 h. For laser irradiation groups, 655 nm laser (0.2 W/cm^2) was employed to irradiate the cells for 10 min. All the cells were further cultured for another 6 h. Subsequently, the cells were washed with PBS thrice and treated with Annexin V-FITC/PI for 20 min. Then the cells were analyzed by flow cytometry.

Tumor Models Establishment.

All animal experiments were conducted and agreed with the Principles of Laboratory Animal Care (People's Republic of China) and the Guidelines of the Animal Investigation Committee, Biology Institute of Shandong Academy of Science, China. Nude mice (6 weeks old, female, ~16 g) were fed with normal conditions of 12 h light and dark cycles and given access to food and water ad libitum. 1×10^7 MCF-7 cells were injected subcutaneously into the right axillary region of the nude mice. After the tumor size had reached approximately 75-100 mm^3, the mice were used in subsequent experiments.

In Vivo Imaging Assay.

For the in vivo imaging assay, 5 μL of COF-survivin (100 μg/mL) was injected into the tumor tissue. For comparing the specificity of the probe, 5 μL of COF-survivin (100 μg/mL) was also injected into the leg tissue. 4 h later, the mice were imaged with a live body imaging system.

In vivo antitumor experiment.

The tumor-bearing nude mice were divided into 4 groups: I: PBS only, II: PBS+Laser, III: COF-survivin, IV: COF-survivin+Laser. For COF-survivin containing groups, 20 μL of COF-survivin PBS solution (1 mg/mL) was injected into the tumor tissue of the mice. 4 h later, the mice in laser irradiation groups were treated with 633 nm laser (0.2 W/cm^2) for 20 min. After that, the tumor growth and body weight change situations of the mice were recorded within 14 days. For H&E staining assay, at the 3rd day, the tumor tissue and the main organs of liver, heart, spleen, lung, kidney collected for H&E staining. For the survival rate, the mice were identified as death as the tumor volume reached 500 mm^3.

Prognostic evaluation of antitumor effect.

For evaluating the prognostic effect of COF-survivin, at the 7th day after PDT, 5 μL of COF-survivin (100 μg/mL) was subcutaneous injected into the treated tumor tissue. 4 h later, the mice were imaged with a live body imaging system.

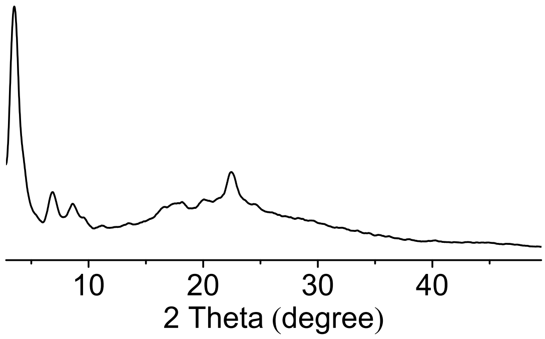

Figure S1. The XRD pattern of COF.

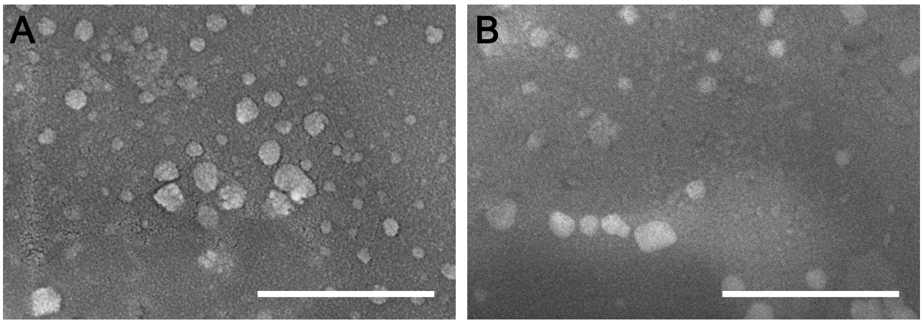

Figure S2. SEM images of (A) COF NPs and (B) COF-survivin. The scale bars are 500 nm.

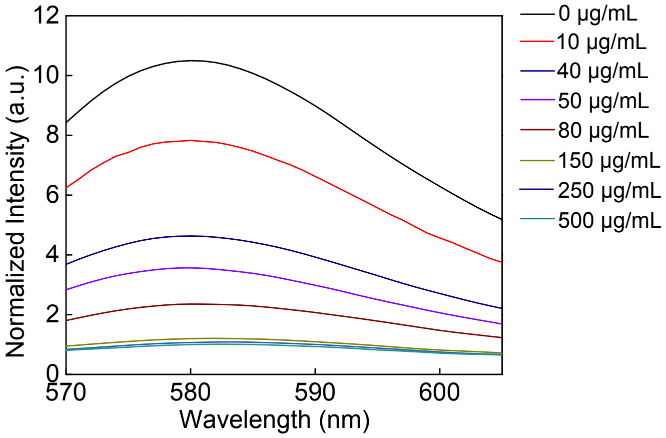

Figure S3. Fluorescence quenching of the TSAS (50 nM) with various concentrations of COF NPs. Ex = 555 nm, Em = 580 nm.

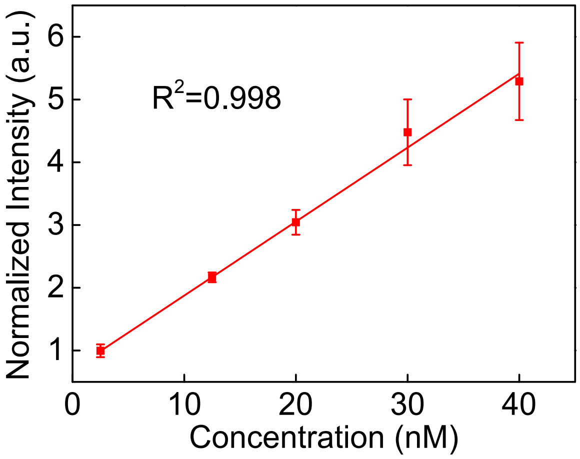

Figure S4. Fluorescence standard curve of TSAS solution. Ex = 555 nm, Em = 580 nm.

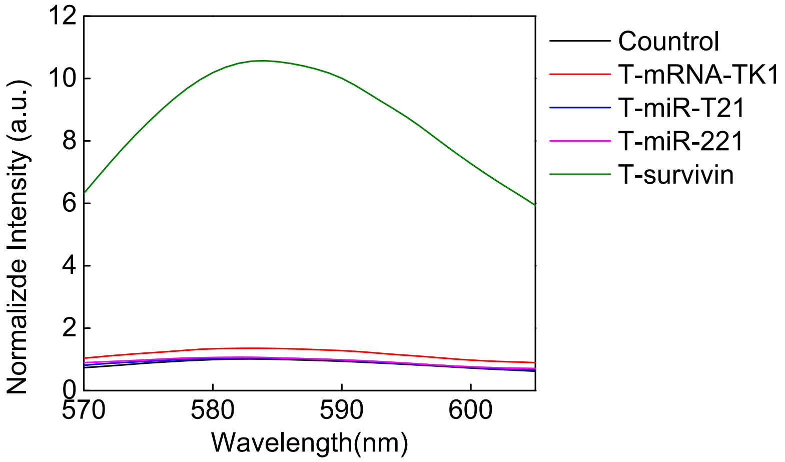

Figure S5. Fluorescence signal changes of COF-survivin in the presence of various targets (500 nM), COF-survivin solution was used as the control. Ex = 555 nm, Em = 580 nm.

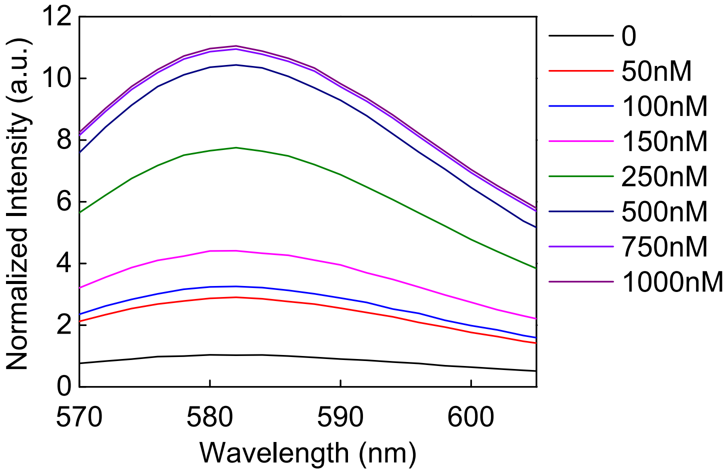

Figure S6. Fluorescence signal changes of COF-survivin in the presence of various concentrations of survivin target. Ex = 555 nm, Em = 580 nm.

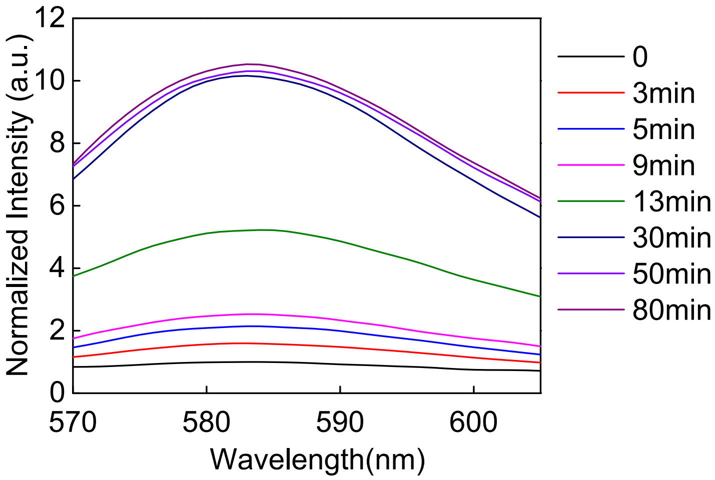

Figure S7. Time-dependent fluorescence signal changes of COF-survivin in the presence of survivin target (500 nM). Ex = 555 nm, Em = 580 nm.

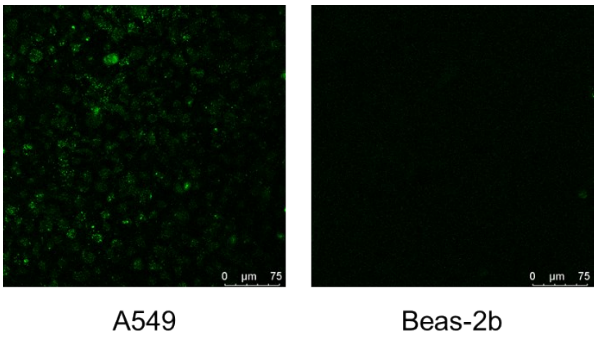

Figure S8. Confocal images of A549/Beas-2b cells treated with COF-survivin. Scale bar = 75 nm.

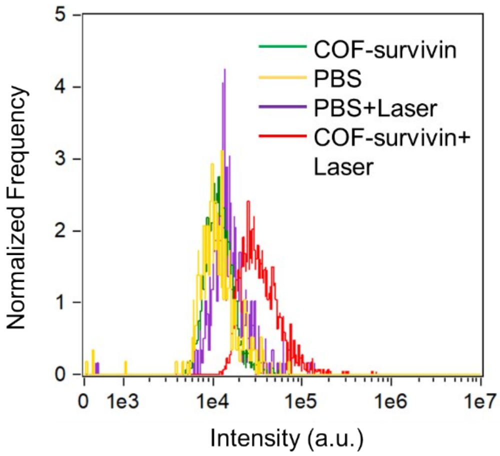

Figure S9. Flow cytometry analysis of ROS accumulation in MCF-7 cells with different treatments.

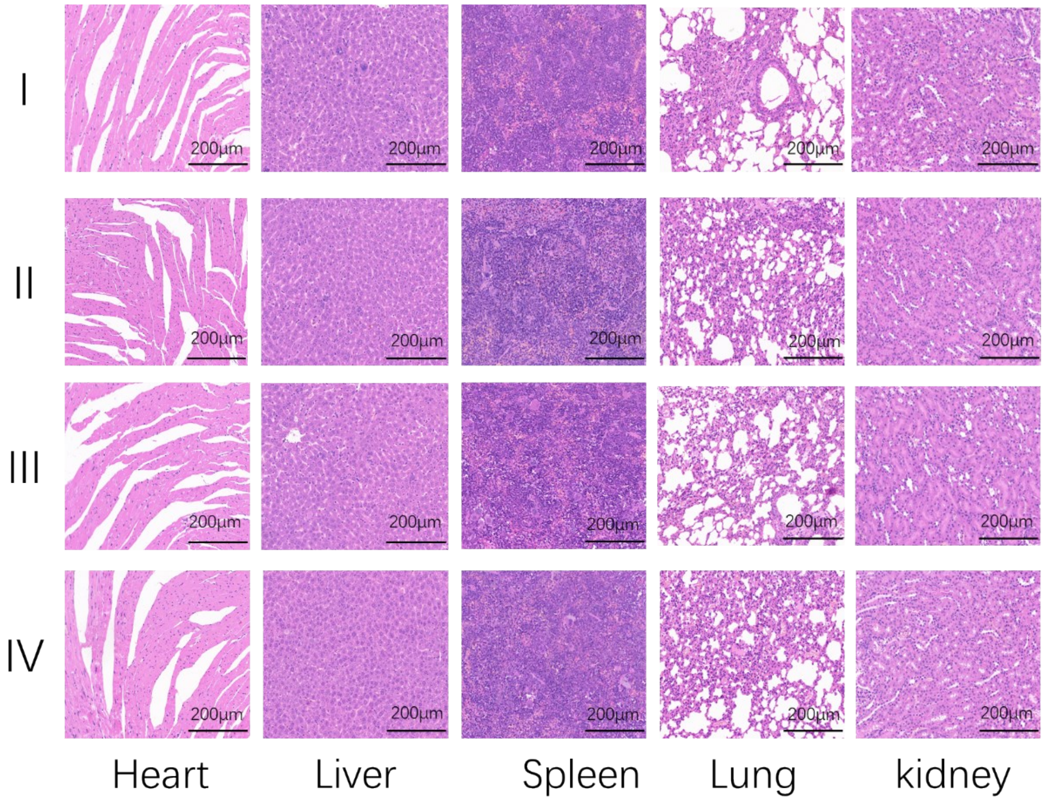

Figure S10. H&E staining of the main organs of mice with different treatments. I: PBS only, II: PBS+Laser, III: COF-survivin, IV: COF-survivin+Laser.

Table S1. DNA sequences employed in this work.

Oligonucleotide	Sequence (5′-3′)
**TSAS**	TAMRA-TAGAGATGCGGTGGTC
**T-miR221: miRNA-221 target**	AGCTACATTGTCTGCTGGGTTTC
**T-miR21: miRNA-21 target**	TAGCTTATCAGACTGATGTTGA
**T-mRNA TK1: TK1 mRNA target**	AAGTATGCCAAAGACACTCGC
**T-survivin: survivin mRNA target**	GACCACCGCATCTCTA
